# Editorial: Advances in Parkinson's Disease Research: Exploring Biomarkers and Therapeutic Strategies for Halting Disease Progression

**DOI:** 10.3389/fnagi.2025.1640566

**Published:** 2025-06-27

**Authors:** Anastasia Bougea, Yildiz Degirmenci

**Affiliations:** ^1^Department of Neurology Medical School of Athens, National and Kapodistrian University of Athens, Athens, Greece; ^2^Neurology Department, Istanbul Health and Technology University (ISTUN), Zincirlikuyu Medicana Hospital Neurology ClinicParkinson's Disease and Movement Disorders Unit, Istanbul, Türkiye

**Keywords:** biomarkers, treatments, Parkinson's disease (PD), risk factors, bibliometric analysis

## 1 Introduction

Parkinson's disease (PD) is a multifactorial neurodegenerative disorder, characterized by loss of dopaminergic neurons of substantia nigra (SN) in 1% of people aged above 65 years (Ben-Shlomo et al., 2024). Its complex clinical picture includes motor symptoms such as tremor, bradykinesia, and gait instability, as well as non-motor symptoms (depression, psychosis, cognitive decline) (Schilder et al., 2017; Titova and Chaudhuri, 2017). Current symptomatic therapies have limited long-term efficacy (Aldaajani and Khalil, 2024). A deep analysis of neural network after PD onset could deepen our understanding of the molecular crosstalk and biological processes underlying PD pathogenesis (Tomkins and Manzoni, 2021). However, there is a lack of reliable biomarkers for early diagnosis, presenting barriers to monitoring and developing disease-modifying therapies.

This Research Topic of Frontiers in Neurology entitled “Body Fluid Biomarkers in Neurodegenerative Studies: Novel Insights into Pathophysiology to Support Clinical Practice and Drug Development” includes articles on innovative biomarkers and interventions.

## 2 Risk factors of Parkinson's disease

This Research Topic includes three studies that examine different factors that influence the PD progression (Paul et al., 2019).

Wei et al. showed that age, gender, smoking, history of falls, hypertension, diabetes, and poor physical activity are risk factors of postural instability of PD patients.

According to Huang et al., the Composite Dietary Antioxidant Index (CDAI) is an independent risk factor for PD risk in the general population and all-cause mortality in PD patients. These findings strength the development of tailored antioxidant-boosting dietary therapies for both established PD patients and high-risk groups with inadequate CDAI.

He et al. support HLA-DRB1 rs660895-G allele as a protective genetic factor for PD risk in Chinese population. Furthermore, authors provide new evidence for the protective effect of rs660895-G allele in PD progression.

These findings focus on managing life style risk factors and promoting light physical activity to improve balance and reduce fall risks. CDAI criteria enable targeted nutritional therapies, emphasizing antioxidant-rich dietary advice for patients with lower PD risk. Understanding of the genetic factors underlying PD in the Chinese population offers a new gene target for therapeutic development.

## 3 Biomarkers of PD: advances in detection

There are several different types of biomarkers for PD, such as clinical, neurochemical, and genetic (Bougea, 2020). Ten studies provided novel insights into the early detection and monitoring of PD.

Sun et al. found that facial expression-induced enhancement as measured by P1 amplitude in the occipital region of the PD group can be used as a rapid and objective test to screen for depressive symptoms in PD.

Zhang J. et al. identified circadian rhythm genesAK3, RTN3, and LEPR as biomarkers in the progression of PD by regulating NK cells, however, the exact mechanism is not clear.

Wang Q. et al. confirmed that GPR78, CADM3, and CACNA1E were biomarkers that mostly participated in pathways, such as the “cell cycle” and “hydrogen peroxide catabolic process.” They also found; five types of immune cells that differed between PD and control groups.

Zhang F.-L. et al. highlighted the potential value of oligodendrocyte precursor cells (OPCs), AGPAT4, DNM3, PPP1R12B, PPP2R2B, and LINC00486as biomarkers in PD progression.

Wang S. et al. observed significant distinctions in voxel-wise fractional amplitude of low-frequency fluctuation (fALFF) marker of the spontaneous brain activity within cerebellum and cortices of early stages of MSA-P and PD.

According to their two-sample bidirectional Mendelian randomization (MR) analysis Lv et al. did not identify significant associations of oxidative stress biomarkers such catalase, glutathione peroxidases, superoxide dismutase, vitamins A-C-E-B12, folate, copper, or iron with PD.

Hu et al. suggested that longer anticipatory postural adjustment (APA2) duration was associated with increased risk of falls, as a potential biomarker for disease progression.

According to Yusufujiang et al. cathepsin B exert a neuroprotective effect and could be used as a predictive biomarker for PD vulnerability, offering novel perspectives into the disease's etiology and potential treatments.

Wang M. et al. marked and optically scanned blood vessels and astrocytes in the SN area of PD mice in order to investigate the alterations in the neurovascular unit (NVU) in the SN area. The SN region's glial cell, dopaminergic neuron, and blood vascular interconnection, together with modifications in their spatial positional correlations, may function as a biomarker of PD progression.

By using aceRNA regulatory network, Chun and Kim suggested 36 plasma lncRNAs as diagnostic biomarkers involved in PD pathogenesis, which regulate gene expression in the SN of the brain.

Together, these studies highlight the importance of combination of biomarkers and risk variables into predictive models, improving early diagnosis and monitoring of PD. Sme of them may serve as diagnostics (lncRNAs, P1 amplitude) or predictive (NVU, cathepsin B, APA2, and circadian rhythm genesAK3) may shed novel light on the pathogenesis of PD.

## 4 Innovative therapeutical approaches for PD

Both pharmaceutical and non-pharmacological (cognitive training, physical activity, and dietary changes) treatments are used to treat PD symptoms (Degirmenci et al., 2023; Ernst et al., 2024). Seven promising approaches were also highlighted by this Research Topic.

Wu Z. et al. suggests manual acupuncture combined with antidopaminergic treatment may ameliorate anxiety in PD patients, but low quality RCTs and the lack of significant effects of electroacupuncture warrant more prospective studies.

Fu et al. offer preliminary insights into the mechanism of transcutaneous auricular vagus nerve stimulation (taVNS) in treating PD. TaVNS appears to reduce whole-brain amplitude of low-frequency fluctuations in specific brain regions, suggesting a potential modulation mechanism for treating PD.

HongFei et al. found that archery, cycling, and dual rhythm dance are effective for motor function in individuals with early- to middle-stage PD. Their study provided a reference for healthcare practitioners in choosing exercise programs to improve cardiovascular health and quality of life.

Izquierdo-Altarejos et al. showed that Golexanolone, a well-tolerated GABAA receptor-modulating steroid antagonist (GAMSA), may be useful in improving motor incoordination, fatigue, anxiety, depression, and short-term memory. Notably, golexanolone reduced the activation of microglia and astrocytes, decreased TH loss 5 weeks postoperatively, and prevented the increase of a-synuclein levels at 10 weeks.

Feng et al. suggested fecal microbiota transplantation (FMT) as a potential PD treatment. FMT can rebuild the gut microbiota by alleviating oxidative stress, thereby reducing motor and non-motor symptoms of patients with PD.

Ullah et al. found that nicotine increased neuroprotection and reduced α-synuclein accumulation in 6-OHDA-treated nematodes. This natural compound also boosted lipid deposition, food sensing behavior, SOD-3, and Daf-16 fluorescence. Zhang Y. et al. showed that acupuncture, cognitive behavioral therapy, exercise and repetitive transcranial magnetic stimulation significantly improved sleep, depression, anxiety, cognition, constipation, and quality of life of PD patients.

Studies suggest personalized pharmaceutical and non-pharmacological therapies for PD, with nicotine, Golexanolone, taVNS, acupuncture, and FMT showing promising antiparkinsonian properties, by modulating brain activity. Further research is needed to validate their sustainability, safety, and effectiveness.

## 5 Applying bibliometric analysis to reveal current research

Bibliometric analysis is a systematic approach to evaluating scientific literature and detecting patterns, and effects by using quantitative tools to filter data from relevant sources (Passas, 2024). This Research Topic includes two bibliometric studies that significantly expand their respective fields.

Wang Y. et al. conducted the first comprehensive bibliometric analysis of PD transcriptomics research. Research hotspots including novel machine learning applications and biomarkers demonstrate advances in translational science. However, poor biomarker validation is a major limitation.

Wu J. et al. performed a bibliometric analysis to investigate the research hotspots on PD related psychosis (PDP), such as pimavanserin, risk factors, and functional connectivity. Novel 5-HT2A receptor inverse agonists and mGlu2 agonists are being thoroughly examined in the field of new drug development.

## 6 Conclusion

This Research Topic combines important studies on the risk factors, treatments, biomarkers, and bibliometric analysis. The results of these studies provide a useful guide for clinicians in their practice and to suggest targets for researchers in developing new diagnostics and therapeutic strategies.
